# Conversion of Ethanol to Guerbet Alcohols and Other Products: Combined Catalytic and Theoretical Study

**DOI:** 10.1021/acsomega.5c05882

**Published:** 2025-12-10

**Authors:** Jan Malina, Karel Frolich, Martin Hájek, Jaroslav Kocík, Vladimír Lukeš, Erik Klein

**Affiliations:** † 48252 University of Pardubice, Faculty of Chemical Technology (Department of Physical Chemistry), Studentská 573, 532 10 Pardubice, Czech Republic; ‡ 223693 ORLEN Unipetrol RPA s.r.o., Záluží 1, 436 70 Litvínov, Czech Republic; § Institute of Physical Chemistry and Chemical Physics, Slovak University of Technology in Bratislava, Radlinského 9, Bratislava SK-812 37, Slovakia

## Abstract

The present study investigates the catalytic upgrading of bioethanol to higher-value chemicals through heterogeneous catalysis, with a focus on the production of 1-butanol as a versatile compound widely used in many applications. Copper-impregnated Li-Al mixed metal oxides were synthesized and characterized as catalysts for ethanol transformation. Comprehensive characterization techniques were involved to correlate catalyst properties with their performance and describe catalyst stability. The catalysts showed promising activity in the formation of higher alcohols under a high weight hourly space velocity (WHSV) and pressure. Theoretical calculations were incorporated into the study to describe the feasibility of individual steps in the Guerbet reaction with main side reactions involved and to supplement information regarding the influence of the catalyst itself. The temperature effects on the reactions were illustrated. In this way, we attempted to explain the role of the catalyst in the reaction pathways, contributing to a more precise tuning of the catalytic system.

## Graphical abstract

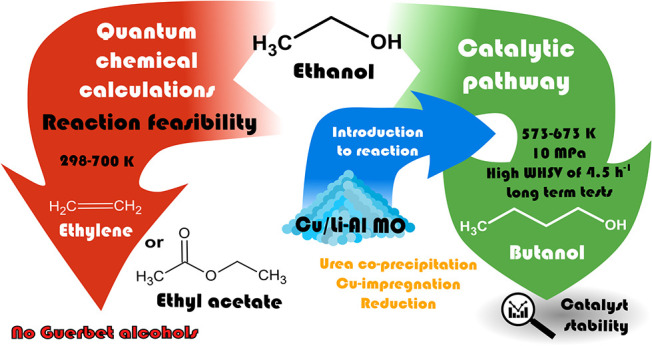

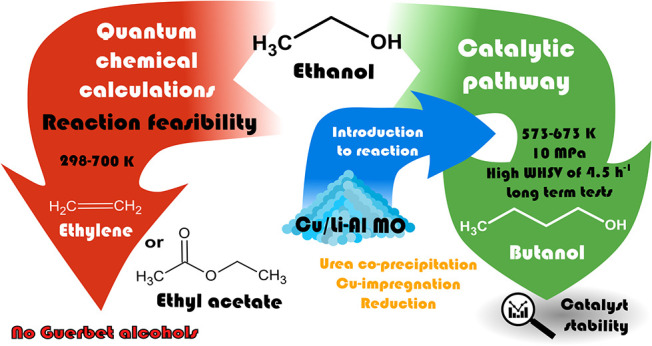

## 1 Introduction

Ethanol is a widely available and economically viable feedstock that can be produced sustainably from renewable resources such as lignocellulosic biomass and biowaste.^1^ Its production is well established, and its high availability makes it convenient feedstock for upgrading into not only other bulk chemicals but also products with higher added value. Beyond its direct use as a fuel or solvent, ethanol serves as a promising platform molecule in the development of advanced biobased chemicals. Higher alcohols could be found among the various products with higher added values obtainable from ethanol, primarily 1-butanol, which has a wide variety of usage. It can be used as a fuel or a fuel additive,^2^ where its properties surpass ethanol due to the higher energy content, lower hygroscopicity, and better miscibility with gasoline compared to ethanol.^3^ In addition to its role as a potential biofuel, 1-butanol is an important precursor in the chemical industry, where it is utilized in the production of solvents (butyl acetate, etc.), polymers (butyl acrylate, etc.), plasticizers (butyl phthalates, etc.), and preservatives (butylparaben, etc.).^4^ Similarly, esters derived from ethanol or higher alcohols offer important applications in fragrances, solvents, and biolubricants.^5^ Thus, upgrading ethanol to referred compounds represents a highly attractive route not only in the context of sustainable chemistry but also to lower the prices of final products.

Conversion of ethanol to higher alcohols can be achieved through various catalytic processes. One of the most investigated methods for producing 1-butanol is the Guerbet reaction,^6^ which involves a reaction sequence of dehydrogenation, aldol condensation, dehydration, and hydrogenation steps.^6–8^ These reaction steps require an efficient catalyst. Several homogeneous molecular bifunctional catalysts with Ru, Ir, and Mn were selected for their ability in hydrogen transfer reactions and revealed efficiency in ethanol upgrade to higher alcohols in the liquid phase.^9–11^ Nevertheless, homogeneous catalysts are related to the problem of their separation from reaction mixtures, which makes their recycling difficult and, therefore, very expensive for industrial applications. In addition, molecular catalysts tend to be sensitive to air, moisture, and temperature, which leads to degradation and loss of activity. Heterogeneous catalysts offer multifunctionality, tunability, relative stability, and reusability.^12^ Various heterogeneous catalyst systems have been explored, including hydroxyapatite, which showed limited activity,^13^ and magnesium oxide, which demonstrated significantly higher activity and thus was chosen for further research.^14^ Mg-Al mixed oxides based on layered double hydroxides (LDHs) gained attention also due to their variable acid–base properties and reasonable performance.^15^

Further improvements were achieved by doping various mixed metal oxides with transition metals (Cu, Co, Ni, etc.), enhancing the dehydrogenation step due to their redox activity.^7,12,16^ Supported metal catalysts, such as Cu/Ni on Al_2_O_3_ or Cu on CeO_2_-activated carbon, have also been investigated with promising results.^17^ In such catalysts, transition metals act as the active phase in dehydrogenation steps and the oxide support acts as the active phase in aldol condensation and dehydration. With Mg-Al mixed oxide systems approaching their performance limits, attention has shifted to alternative compositions such as Li-Al mixed oxides, which exhibit structural features with additional potential for modification.^18^ In particular, Cu/Li-Al catalysts relate to the potentially higher density of strong basic sites, and according to our previous results,^7,19,20^ lower Cu loading is needed to obtain comparable ethanol conversions to Cu/Mg-Al at the same catalytic conditions.

Research on the Guerbet reaction has progressed significantly, and a wide range of catalytic results have been reported, which are primarily attributed to the catalyst structure. However, different reaction conditions have been used, making a direct comparison of catalysts rather difficult. The question arises why side reactions occur and what is their feasibility and how are the individual steps of the Guerbet reaction and the side reactions affected by the temperature. Here, theoretical calculations are implemented to gather insight into the individual steps in the Guerbet reaction and main side reactions in terms of thermodynamics. The experimental part focuses on Li-Al mixed oxides impregnated with copper for Guerbet-type ethanol condensation to higher alcohols. The previously optimized Li-Al oxide matrix^19^ is loaded with different amounts of copper. Long-term catalytic tests (136 h each) are carried out in a fixed-bed microflow reactor to obtain reliable catalytic data and to study the catalyst stability at a high WHSV. The catalyst structure and redox behavior are related to the catalytic performance, particularly ethanol conversion and product selectivity. The novelty lies in the combination of catalytic and theoretical approaches, which offers a better understanding of the reaction catalytic process, foreshadowing the future path in the catalytic research of the Guerbet reaction.

## 2 Methods

### 2.1 Catalyst Preparation and Treatment

The optimized urea synthesis method was used to prepare the LDH precursor of the porous Li-Al metal mixed oxide. The detailed synthesis conditions were described in previous studies.^19–21^ The synthesis targeted the highest Li content in the LDH which corresponded to a maximum Li:Al ratio of 0.5:1. A stoichiometric excess of Li was used in the LDH precipitation; specifically, the nitrates were mixed with respect to the Li/Al molar ratio of 3:1. Basic pH was maintained by addition of urea, where the urea/nitrate ratio was at 3:1. The precipitation proceeded at 105 °C for approximately 24 h. After that, the LDH precursor was filtrated and then calcined at 550 °C to obtain a mixed oxide. The Li-Al mixed metal oxide was further subjected to copper impregnation.

The obtained Li-Al oxide matrix was transferred into a 250 mL beaker with 100 mL of copper nitrate solution, which was stirred with a magnetic stirrer for 24 h. In the next step, excess water was evaporated at 373 K. The concentrated suspension was obtained, transferred to the Petri dish, and placed into the oven, where it was left to dry at 333 K for 24 h. In the final step, the impregnated mixed oxide was placed into the oven and calcined at 873 K to obtain the final form of the Li-Al mixed metal oxide with copper oxide, denoted as a fresh catalyst.

After the catalytic tests, the catalysts were subjected to subsequent treatments in the form of calcination in an air atmosphere to burn out the potential carbon residues on the catalysts. The calcination was done at 673 K for 4 h with a heating ramp of 10 °C min^–1^. The catalysts in this form were denoted as spent catalysts and were characterized by the same methods as used for fresh catalysts.

### 2.2 Analytical Methods

Both forms of catalysts, fresh and spent, were described by the following characterization methods.

Inductively coupled plasma mass spectrometry (ICP-MS) was utilized to determine the actual molar ratios of metals present in the catalysts. The analysis was performed using a 7900 ICP-MS instrument (Agilent Technologies).

X-ray diffraction (XRD) was utilized to confirm successful catalyst synthesis and to investigate the crystallographic structure. Measurements were carried out using a D8 Advance diffractometer (Bruker AXS GmbH) equipped with a Cu Kα radiation source and a secondary graphite monochromator. Data were collected over a 2θ range of 1–90° with a step size of 2°.

Morphological and elemental analyses were conducted using scanning electron microscopy coupled with energy-dispersive X-ray spectroscopy (SEM-EDX). A TM 4000Plus tabletop SEM instrument (Hitachi, Japan) was employed, operating under high vacuum with a 10 kV electron beam.

Nitrogen adsorption isotherms were measured by using the ASAP 2020 system (Micromeritics) at 77 K to characterize the textural properties of the calcined materials. Specific surface areas were calculated using the Brunauer–Emmett–Teller (BET) method, while the pore size distribution, volume, and diameter were derived using the nonlocal density functional theory (NLDFT) model.

The reducibility of copper species in the catalysts was examined by H_2_ temperature-programmed reduction (H_2_-TPR), while surface acidity and basicity were evaluated through NH_3_ and CO_2_ temperature-programmed desorption (NH_3_-TPD and CO_2_-TPD), respectively. These experiments were conducted using the AutoChem II 2920 chemisorption analyzer (Micromeritics) equipped with a thermal conductivity detector (TCD) for TPR and coupled with an OmniStar GSD 320 mass spectrometer (Pfeiffer Vacuum, Germany) for TPD measurements. Approximately 100 mg of the catalyst was used for each experiment. High-purity gases were used: O_2_ (99.5%), He (99.9999%), H_2_ (99.9999%), CO_2_ (99.9999%), and NH_3_ (99.9999%).

The standard TPR procedure involved pretreatment at the relevant calcination temperature under an oxygen atmosphere, followed by cooling to the reduction starting temperature (303 K) and initiating reduction under a hydrogen atmosphere with a temperature ramp to 823 K. TPD experiments involved pretreatment under an inert atmosphere (He), cooling to the adsorption temperature, saturation with CO_2_ or NH_3_, inert gas purging (He), and temperature-programmed desorption. Both TPR and TPD were performed by using a heating rate of 10 K min^–1^ and a gas flow rate of 25 cm^3^ min^–1^.

Further details of the characterization methodologies are available in our previous studies.^20,22,23^

### 2.3 Calculations

The reaction Gibbs energy (ΔGr) for all reaction steps^7^ was calculated at temperatures of 298, 400, 600, and 700 K using a theoretical approach, as the enthalpy, entropy, and molar heat capacity for all reaction intermediates and products are not available in the literature. These computations were performed with the Gaussian 16 program suite,^24^ employing the hybrid M06–2× functional^25^ for geometry optimizations of reactants and products without any constraints. The calculations were conducted with an energy cutoff of 10^–5^ kJ mol^–1^ and a final RMS energy gradient below 0.01 kJ mol^–1^ Å^–1^. This function is considered a reliable choice for thermochemical calculations across a wide range of organic compounds.^25,26^ The 6–311++G­(d,p) basis set,^27,28^ incorporating diffuse and polarization functions, was selected to ensure a balanced description of all molecules under study. Reaction enthalpies and Gibbs energies for individual reaction steps were derived from the calculated total enthalpies and Gibbs energies at 298, 400, 600, and 700 K.

The accuracy of the calculations was evaluated for homogeneous gas-phase thermochemical properties of reactions involving the studied compounds. For selected reactions, the reaction enthalpies at 298 K were compared with values derived from the formation enthalpies of reactants and products reported in the Active Thermochemical Tables^29^ (based on highly precise experimental measurements with uncertainties not exceeding 0.4 kJ mol^–1^). The results validated the applicability of the chosen computational methodology (Table S1).

### 2.4 Catalytic Tests

The catalytic tests were carried out in a microreactor (Multireactor catalyst testing unit, Vinci Technologies, France). 1.5 g of catalysts were mixed with inert silicon carbide to ensure a constant volume of the catalyst bed. Coarser silicon carbide was also used to secure the main catalyst bed in the reactor. The catalyst was activated in a stream of hydrogen (10 dm^3^ h^–1^) at 773 K and 1.5 MPa for 5 h. The reaction was started after activation by introducing the catalyst to 6.75 g h^–1^ of ethanol in a flow of N_2_ (WHSV = 4.5 h^–1^), at 10 MPa pressure and 573 K. Note: the WHSV is defined as the flow of reactant divided by the catalyst mass. This reaction temperature was maintained for 38 h, after which the reaction temperature was changed in the following sequence: 573, 623, and 673 K (heating rate 30 K h^–1^). Each temperature from this sequence was held for 32 h. Samples (liquid and gas) of the reaction mixture were taken every 4 h.

The composition of the reaction mixture (liquid and gas phases) was studied by gas chromatography. The liquid phase was analyzed by the gas chromatograph with flame ionization (GC-FID Shimadzu GC-2010; Lion LN-624 GC 30 m × 0.25 mm × 1.4 μm). Analysis of the gas phase was conducted also by the gas chromatograph (GC-Agilent 7890 A, Agilent Technologies, INC) equipped with three detectors: two thermal conductivity detectors to detect permanent gases (for example, hydrogen) and one flame ionization detector for the detection of hydrocarbons.

The ethanol conversion was calculated by the formula

$$X_{EtOH}=\frac{n_{EtOH,feed}-n_{EtOH,output}}{n_{EtOH,feed}}\cdot 100\;\left(\%\right)$$

The selectivity of product (i) was calculated by the formula

$$S_{i}=\frac{n_{i,output}}{n_{EtOH,feed}-n_{EtOH,output}}\cdot \frac{\nu_{EtOH}}{\nu_{i}}\cdot 100\;\left(\%\right)$$

In the formulas, *n*_EtOH_ and *n*_i_ denote the molar amounts of *ethanol* and the *i*th product, respectively, and *v*_EtOH_ and *v*_*i*_ denote the stoichiometric coefficient of *ethanol* and the *i*th product, respectively.

## 3 Results and Discussion

### 3.1 Catalyst Characterization

The following chapters are focused on the characterization of catalysts used for the Guerbet reaction in their fresh (before the reaction) and spent (after the reaction) forms. The observed properties will be used for the correlation with catalytic results for a deeper understanding of the catalyst performance.

#### 3.1.1 ICP-MS

The chemical composition of Li-Al mixed metal oxides impregnated with copper was investigated using ICP-MS analyzing the weight percentage of metals (Table 1). The molar ratio of Li:Al detected in the range 0.43:1 to 0.41:1 corresponded to the same Li-Al parental matrix for catalysts. The real copper content was a bit higher than the theoretical amount used for impregnation, but the deviation among theoretical and real copper contents was detected only 0.6% at maximum. To distinguish among the catalysts, the nominal copper content (0.5, 5, and 10 wt %) was used in the nomenclature of oxides.

**Table 1:** Properties of Cu/Li-Al Mixed Metal Oxide Catalysts

			(Cu:Al)_surf_ ^a^	(O:Al)_surf_ ^a^	*S*_BET_, m^2^ g^–1^	pore volume, cm^3^ g^–1^	CBS, μmol g^–1^	CAS, μmol g^–1^	H_2_ consumption^b^, cm^3^ g^–1^
nomenclature	Cu, wt %	molar ratio Cu:Li:Al	F/S^c^	F/S	F/S	F/S	F/S	F/S	F/S
Cu_0.5	0.52	0.005:0.43:1	0.04/0.01	1.90/1.97	119/114	0.619/0.590	341/153	404/206	1.77(1.93)/0.99(1.08)
Cu_5.0	5.06	0.05:0.42:1	0.08/0.07	2.11/2.69	100/97	0.588/0.571	283/179	336/161	12.64(1.42)/14.06(1.58)
Cu_10.0	10.58	0.10:0.41:1	0.19/0.10	2.08/2.69	90/91	0.431/0.430	215/139	277/123	31.01(1.66)/27.65(1.48)

a From EDX analysis.

b The number in the parenthesis is the average change of the copper oxidation number.

c F/S denotes fresh and spent catalyst forms.

#### 3.1.2 XRD

XRD was used to confirm the successful synthesis of catalysts (fresh form) and effects of the catalytic reaction on the catalyst structure (spent form) (Figure 1).

**Figure 1:**
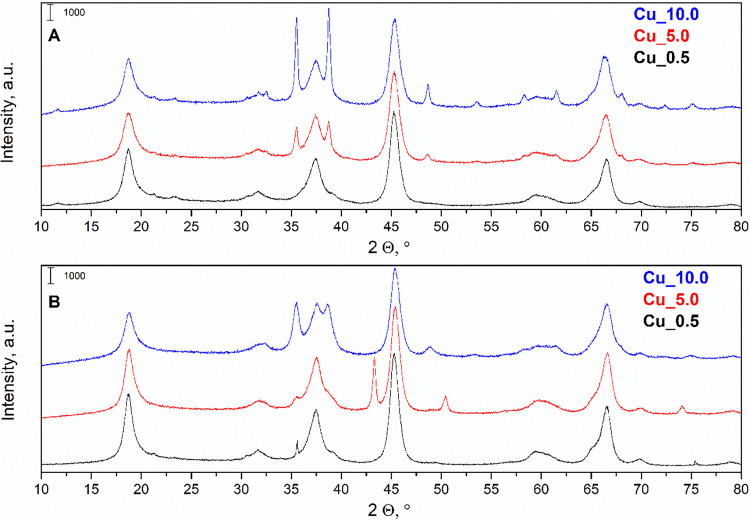
X-ray diffraction patterns of Cu_0.5, Cu_5.0, and Cu_10.0: (A) fresh and (B) spent catalysts

For the fresh catalysts (Figure 1A), the following diffraction lines at 2 Θ ≈ 18.66, 37.33, 45.28, 59.41, and 66.60° were present, which corresponded with the observation of Li-Al mixed metal oxides.^20,30^ The diffraction lines at 2 Θ ≈ 37.33, 45.28, 59.41, and 66.60° also corresponded with the γ–Al_2_O_3_ XRD pattern,^31^ which confirms the theory of lithium intercalated in the alumina structure.^18^ Taking into account the amorphous Al_2_O_3_ phase that is invisible by means of XRD, an amorphous structure containing Al_2_O_3_ could also be formed.^32^ However, the relatively constant line width and pattern background suggest a similar content of the amorphous phase along with the Cu loading. The additional diffraction lines at 2 Θ ≈ 35.50, 38.74, 48.60, and 53.50°, with an increase in intensity along with the copper content in the catalyst, were dominant diffraction lines of CuO.^33,34^ The most complex XRD pattern was detected for the Cu_10 catalyst. The primary reason for the variation in the XRD pattern, specifically in the different intensities of CuO diffraction lines, was the difference in copper loading in the Cu/Li-Al mixed oxide (from 0.5 to 10 wt % Cu). Sharp diffraction lines correspond to a high crystallinity of the CuO phase.

The XRD profiles of spent catalysts showed specific changes in the pattern (Figure 1B). The diffraction lines of the Li-Al mixed oxide remained intact, revealing the preservation of the parental Li-Al oxide matrix (2 Θ ≈ 18.66, 37.33, 45.28, 59.41, 66.60°). The reaction changes affected the copper phases, as it was possible to observe the decrease in intensity of appropriate diffraction lines for CuO (2 Θ ≈ 35.50, 38.74, 48.60, 53.50°). An additional difference was observed for Cu_5.0, where the diffraction lines of metallic copper were detected (2 Θ ≈ 43.24, 50.44°). The presence of the metallic copper metal could be caused by its stabilization in the structure of the Li-Al mixed oxide.

#### 3.1.3 SEM and EDX

SEM showed the catalyst’s particle structure and effects related to the copper loading and catalytic reaction (Figure 2).

**Figure 2:**
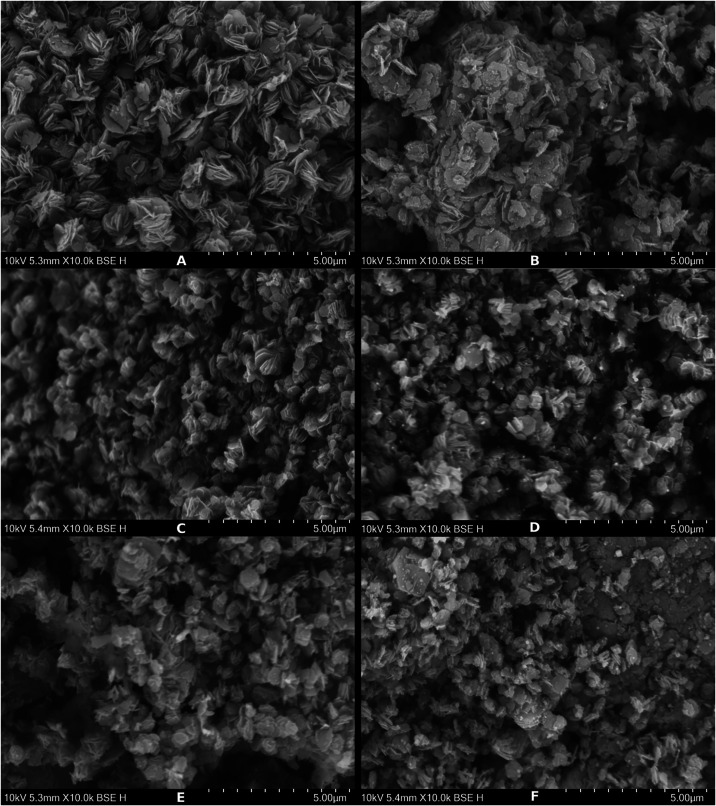
SEM pictures of fresh (on the left) and spent (on the right) catalysts: (A, B) Cu_0.5, (C, D) Cu_5.0, and (E, F) Cu_10.0.

The catalysts in the fresh form showed a well-defined platelike structure, which agreed with the literature.^35^ The influence of copper impregnation was observed, where the defined platelike structure was noticeably decreasing along with the copper loading. All catalysts in the spent form lost their well-defined platelike structure, which appeared less organized and more crumbled. The reaction conditions (673 K and 10 MPa) and reaction mixture were factors of influence in catalyst erosion. A high temperature in combination with other factors also needed to be considered; although fresh catalysts were already exposed to temperatures as high as 773 K during the primary calcination and reduction, one would exclude it from factors of influence in erosion of the catalyst structure.

The EDX analysis revealed the elemental distribution on the surface of particles (outer surface) of fresh and spent catalysts (Figure S1; Table S2).

Aluminum, oxygen, and copper were detected, and for spent catalysts, carbon was also present on the particle surface. The only exception was lithium, which was hard to observe by the EDX method; therefore, lithium was excluded. For the fresh catalyst, the (Cu/Al)_surf_ ratio was markedly higher than the Cu/Al bulk ratio (Table 1), which leads to the conclusion that after impregnation, a significant amount of copper was staged on the surface of the Li-Al oxide particles. This amount relatively decreased with copper loading in the catalyst, i.e., for Cu_10, relatively more copper was incorporated into the porous structure. For the spent catalysts, the (Cu/Al)_surf_ was at lower values, which indicated that during the pretreatment and catalytic reaction, copper migrated into the porous structure of the catalyst. The (O/Al)_surf_ ratios (Table 1) indicated the presence of organic deposits on the surface of the catalyst particles after catalytic tests. Deposits were in relatively higher amounts for Cu_5.0 and Cu_10.

#### 3.1.4 N_2_ Physisorption

The N_2_ adsorption–desorption isotherms (77 K) obtained for fresh catalysts (Figure 3) were all assigned to type IV isotherms, which are typical for the material with a mesoporous structure and are associated with a hysteresis loop. The type of hysteresis loop was assigned as H3, which is typically for platelike particles.^36,37^

**Figure 3:**
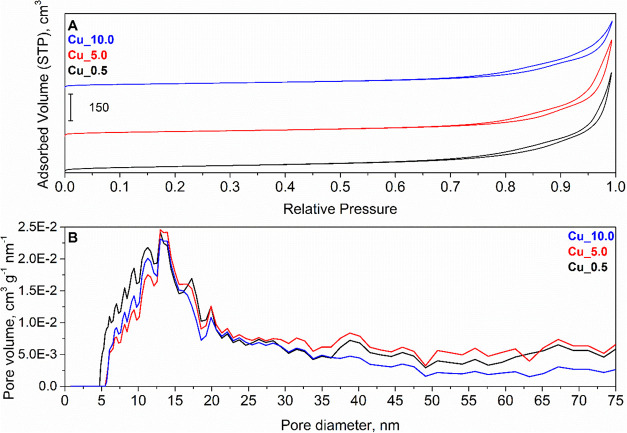
N_2_ physisorption at 77 K on Cu_0.5, Cu_5.0, and Cu_10.0 fresh catalysts. (A) Adsorption–desorption isotherms and (B) pore size distribution.

The specific surface area of fresh catalysts was determined at 119, 100, and 90 m^2^ g^–1^ for Cu_0.5, Cu_5.0, and Cu_10.0, respectively (Table 1), i.e., it decreased with the content of copper impregnated on the Li-Al mixed oxide. This decrease in the specific surface area is one of the disadvantages of preparing mixed oxides using the impregnation method.^38^ The pore size distribution profile was similar for all catalysts with a maximum around 13–14 nm. Only a few minor changes on the distribution profile were observed, such as a shift toward a larger pore diameter for Cu_5.0 and Cu_10.0 compared to Cu_0.5. Catalyst Cu_10.0 was also related to a lower population of larger pores located in macroporous region. However, there was a significant trend of pore volume decrease with copper loading in the Li-Al mixed oxide, being 0.619, 0.588, and 0.431 cm^3^ g^–1^ for Cu_0.5, Cu_5.0, and Cu_10.0, respectively.

The carbonaceous deposits detected by EDX analysis were removed from the spent catalysts by annealing in oxygen before N_2_ physisorption. The N_2_ adsorption isotherms (Figure S2), specific surface areas, and pore volumes (Table 1) of spent catalysts after annealing showed only small changes compared to the fresh catalysts. Although SEM analysis showed a loss of the defined platelike particle structure and EDX analysis revealed copper migration from the outer surface into the porous structure during the catalytic reaction, the texture and pore structure related to only minor changes among the fresh and spent catalysts, which indicated the preservation of the textural properties of the catalyst during the catalytic reaction.

#### 3.1.5 H_2_-TPR

H_2_-TPR was chosen to study redox properties of copper in Cu/Li-Al catalysts in their fresh and spent forms, and the corresponding reduction profiles were obtained (Figure 4). The Li-Al oxide matrix itself is not reduced in the presented temperature interval, i.e., the observed reduction curves are related to the reduction of the supported copper oxides. From the amount of hydrogen consumed during the reduction (area under the curve) and the total amount of copper in the catalyst, the average change in the oxidation state of copper was calculated (Table 1).

**Figure 4:**
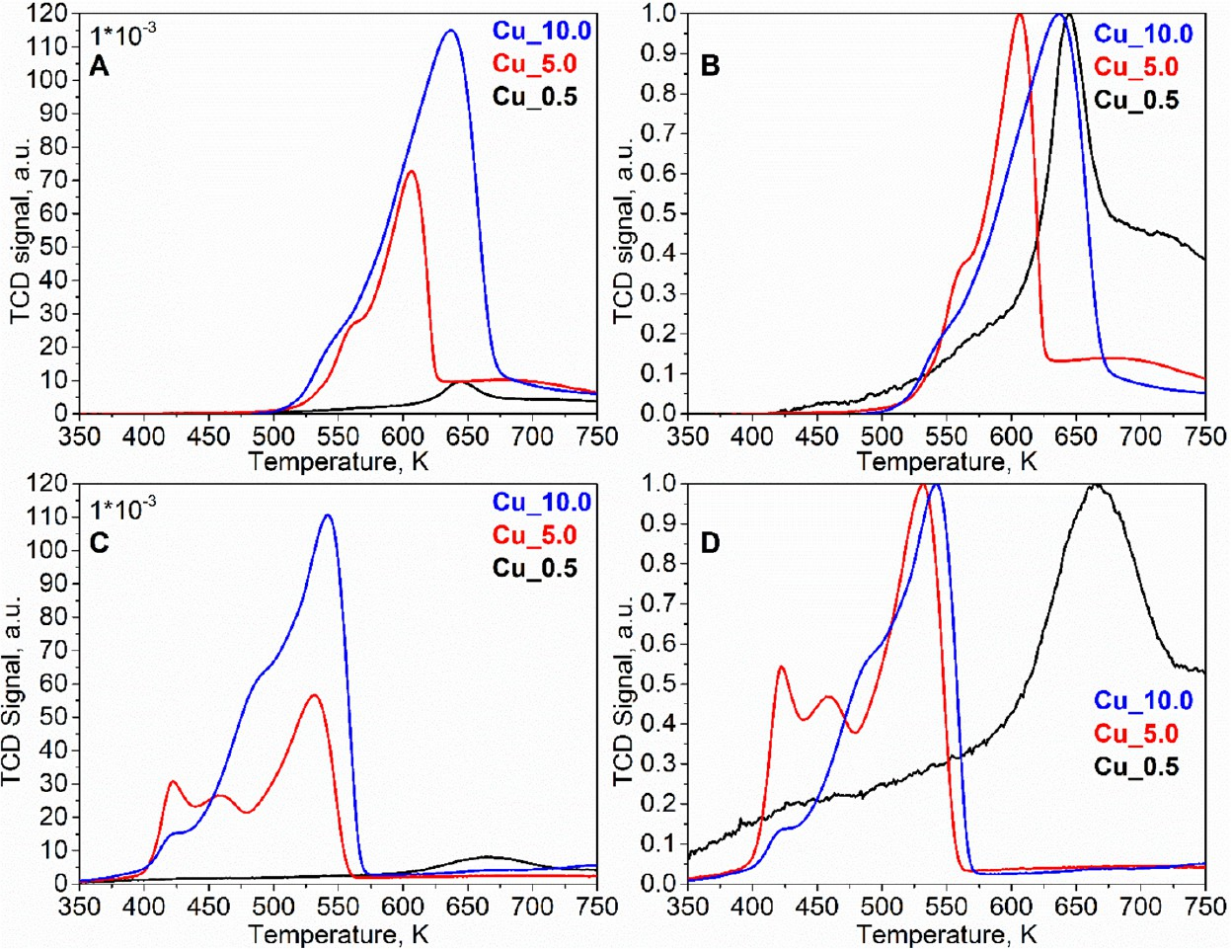
H_2_-TPR reduction profiles of Cu_0.5, Cu_5.0, and Cu_10.0 catalysts: fresh ((A) original and (B) normalized 0–1) and spent ((C) original and (D) normalized 0–1).

The reduction profiles of fresh catalysts (Figure 4A) exhibited distinct maxima at 643, 603, and 633 K for Cu_0.5, Cu_5.0, and Cu_10.0, respectively. The character of the reduction profile was attributed to the level of copper (CuO) impregnation on the Li-Al mixed oxide, as illustrated by normalized curves 0–1 (Figure 4B). The dependence of the reduction rate maxima suggested multiple factors, including the size and stability of copper oxide particles on the Li-Al support. Smaller copper oxide particles usually undergo reduction at lower temperatures but can be effectively stabilized by the support.^39–41^ In the case of Cu_0.5, copper oxide reduction proceeds at a higher temperature compared to the other catalyst, pointing to the stabilization effect of the support. The increased amounts of CuO in Cu_5.0 and Cu_10.0 initiated reduction at lower temperatures due to lower particle stabilization. The primary difference between the Cu_5.0 and Cu_10.0 reduction profiles was the position of the maximum, where Cu_10.0 related to the maximum at a higher temperature, reflecting the reduction of larger CuO bulk particles. The average change in the copper oxidation state reached values of 1.93, 1.42, and 1.66 for Cu_0.5, Cu_5.0, and Cu_10.0, respectively. Values lower than 2 indicate the presence of a portion of copper in the Cu^+^ form.^42,43^

The reduction profiles on spent catalysts characterized the reduction behavior of copper after the catalytic reaction and annealing in oxygen (reoxidation) (Figure 4C). In general, spent catalysts related to a wider interval of Cu reduction and higher Cu heterogeneity. Distinct maxima were identified at 663 K for Cu_0.5 and 543 K for Cu_10.0, whereas Cu_5.0 exhibited three maxima at 418, 458, and 533 K. The average oxidation state changes of copper were calculated to range between 1.08 and 1.57, pointing to a higher abundance of Cu^+^ on spent catalysts. Factors such as temperature (573–673 K), pressure (10 MPa), and the reaction mixture contributed to alterations in the structure of the copper particles; the changes are well illustrated on normalized curves 0–1 (Figure 4D). Observations were related to the migration and redistribution of copper particles, which corresponded to data from EDX analysis. The changes in the reduction profile also corresponded with the XRD diffractogram, where the structure of spent catalysts was less defined and the diffraction lines of CuO were less intensive.

#### 3.1.6 CO_2_- and NH_3_-TPD

The basic and acid properties of catalysts (fresh and spent) were studied by the temperature-programmed desorption of CO_2_ and NH_3_, respectively (Figure 5). The total concentration of Lewis basic sites (CBS) and acid sites (CAS) was obtained from the area under the desorption curve.

**Figure 5:**
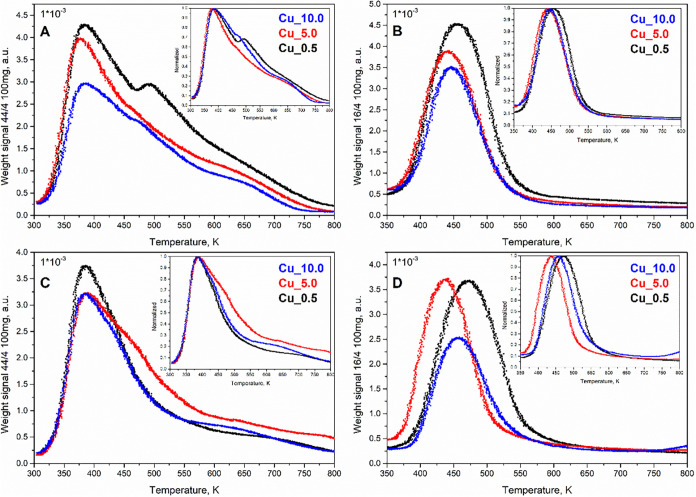
TPD of CO_2_ ((A) fresh and (C) spent) and NH_3_ ((B) fresh and (D) spent) on Cu_0.5, Cu_5.0, and Cu_10.0 catalysts. Insets: normalized curves 0–1.

CBS on fresh catalysts (Table 1) was dependent on copper loading in the catalyst, as Cu_0.5 reached higher CBS (341 μmol of CO_2_ g^–1^) compared to Cu_5.0 (283 μmol of CO_2_ g^–1^) and Cu_10.0 (215 μmol of CO_2_ g^–1^). CAS on fresh catalyst was also dependent on the copper loading (Table 1), as Cu_0.5 reached the highest CAS (404 μmol NH_3_ g^–1^) compared to Cu_5.0 (346 μmol NH_3_ g^–1^) and Cu_10.0 (277 μmol NH_3_ g^–1^). Since the Li-Al matrix from one batch was used for the preparation of the final catalysts, the decrease in CBS and CAS was related to the amount of impregnated copper. Copper partially blocked the acidic and basic sites on the Li-Al oxide surface. It has to be noted that copper oxide itself could provide acidic properties to some extent.^44^

On the CO_2_ desorption curves, three local maxima could be identified (Figure 5A), particularly at 353, 478, and 613 K. The contribution located at 383 K is related to the weak sites, that at 488 K is related to the medium strong sites, and that at 643 K points to the presence of the strong basic sites.^45,46^ The strong Lewis basic sites seem to be crucial for the ethanol condensation into butanol, as it was observed in our recent work.^19^ The impregnation of copper on the Li-Al oxide support did not markedly change the population of individual basic sites (normalized curves insets in Figure 5), i.e., there was no site preferential binding of copper. The NH_3_ desorption curves relate to the symmetrical peak with one maximum at around 458–463 K (Figure 5B), pointing to the presence of one type of Lewis acid site, i.e., acidic sites appeared to be relatively homogeneous.

The spent catalysts were related to the significantly lower values of CBS and CAS than the fresh catalysts (Table 1). Cu_0.5 reached the highest CBS (153 μmol of CO_2_ g^–1^) compared to Cu_5.0 (178 μmol of CO_2_ g^–1^) and Cu_10.0 (139 μmol of CO_2_ g^–1^). Cu_0.5 reached the highest CAS (206 μmol NH_3_ g^–1^) compared to Cu_5.0 (161 μmol NH_3_ g^–1^) and Cu_10.0 (122 μmol NH_3_ g^–1^). The reason for the CBS and CAS decrease was related to the catalyst structure modification during the catalytic reaction. Lower basic and acid site concentrations corresponded with the loss of the well-defined platelike structure observed by SEM. In addition, the parallel with the migration of copper species observed by EDX and H_2_-TPR is pointed out. A change in the individual basic site population on the spent catalyst was observed (Figure 5C), i.e., spent catalysts related to the lower population medium strong and strong basic sites. The NH_3_ desorption curves of spent catalysts (Figure 5D) kept the shape of the well-defined peak pointing to one type of acid sites, i.e., only a small shift in maxima to fresh catalysts was observed.

### 3.2 Reaction Thermodynamics and Catalysis

The following two sections are focused on the study of thermodynamics and catalysis of the Guerbet reaction, presenting the results and discussion of the influence of temperature and the catalyst on the composition of the reaction mixture.

#### 3.2.1 Calculations

The calculation of reaction Gibbs energies, ΔGr, was done for all reaction steps of the Guerbet reaction and selected side reactions of ethanol transformation,^7^ and its values at temperatures of 298, 400, 600, and 700 K are displayed in Figure 6. The ΔGr values were calculated at temperatures that cover the range of the commonly investigated reaction temperatures. ΔGr reveals whether a given reaction is spontaneous (feasible) under given conditions. ΔGr is related to the probability of a given reaction. From the reaction Gibbs energy, it is possible to derive possible pathways where ΔGr is the lowest and thus show probable (intermediate) products of reactions. However, ΔGr does not indicate the rate of reactions and thus does not have a direct connection to catalytic reaction parameters such as conversion and selectivity (yield). Reaction parameters are related to the rate of reactions, which are influenced by the catalyst parameters. Nevertheless, it can be assumed that the lower the ΔGr of a reaction, the higher is the amount of related product(s). Reaction steps with a high positive ΔGr value are considered very unlikely, and the corresponding products should not be present or present only in small amounts, even though the corresponding reaction rates are high. The calculation of ΔGr and its change with temperature help to discuss the role of the catalyst in the reaction and distribution of products. Finding the optimal reaction temperature for the reaction proves to be a significant challenge, particularly if the process should be conducted in a single reactor (a one-step catalytic approach). This complexity comes from the differences in the reaction Gibbs energies of sequential steps of the Guerbet reaction.

**Figure 6:**
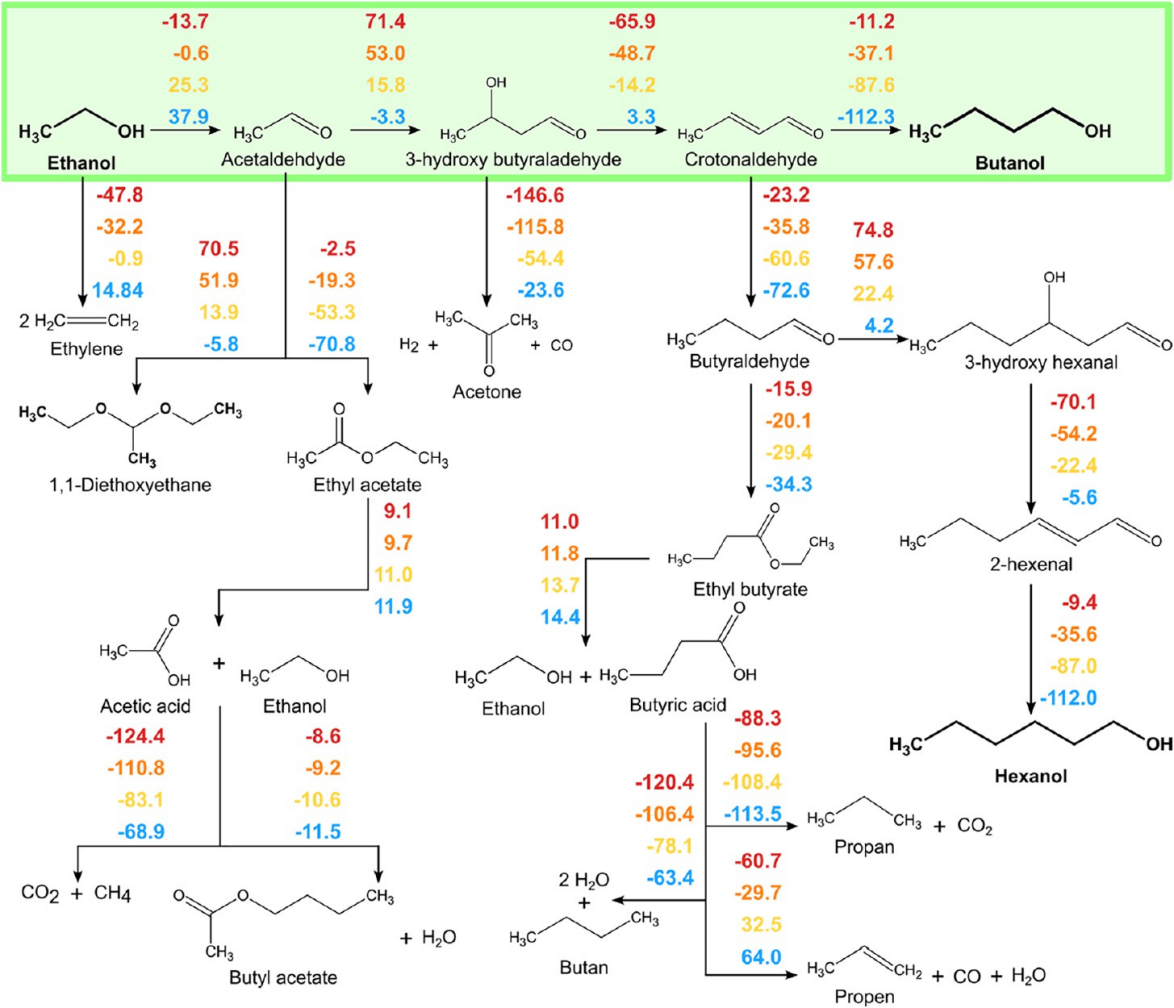
Reaction scheme with reaction Gibbs energies (kJ mol^–1^) for temperatures of 298 K (light-blue), 400 K (yellow), 600 K (orange), and 700 K (red).

ΔGr relates to the feasibility of the individual steps of the Guerbet reaction. The ethanol dehydrogenation step (1st) reached 38 kJ mol^–1^, representing the energetic barrier at 298 K. The following steps of the aldol condensation of acetaldehyde (2nd) and dehydration of 3-hydroxy butyraldehyde (3rd) were related to a ΔGr value around 0 kJ mol^–1^ at 298 K, respectively. On the other hand, the remaining step of crotonaldehyde hydrogenation (4th) reached a ΔGr value below zero, around −112 kJ mol^–1^, representing the most feasible step at 298 K. Each reaction step was related to the change of the ΔGr with the temperature. At 400 K, the decrease of ΔGr was observed for ethanol dehydrogenation (25 kJ mol^–1^) and 3-hydroxy butyraldehyde dehydration (−14 kJ mol^–1^); meanwhile, an increase was observed for the acetaldehyde aldol condensation (16 kJ mol^–1^) and croton aldehyde hydrogenation (−88 kJ mol^–1^). However, ethanol dehydrogenation was the energetic barrier of the Guerbet reaction also at 400 K. Further changes in the ΔGr were observed with the following temperature increase. At 600 and 700 K, ethanol dehydrogenation was related to the decrease in the ΔGr to the values around 0 and −14 kJ mol^–1^, respectively. Dehydration of 3-hydroxy butyraldehyde was also related to the drop in the ΔGr. The ΔGr of acetaldehyde aldolization related to the further increase of its ΔGr, so the aldol condensation step (2nd) was considered as an energy barrier for temperatures over 600 K.

Possible side products which were most likely to occur were ethyl acetate, acetone, and butyraldehyde. Ethyl acetate as another product of acetaldehyde condensation (3-hydroxy butyraldehyde) followed an upward trend with increasing temperature, where it related to an increase in the ΔGr reaching −71 kJ mol^–1^ at 298 K and −3 kJ mol^–1^ at 700 K. The ΔGr of acetone forming from 3-hydroxy butanal followed a downward trend with temperature in the interval from −24 kJ mol^–1^ (298 K) to −147 kJ mol^–1^ (700 K). The ΔGr of butyraldehyde forming was related to the upward trend with temperature since it reached values in intervals from −73 kJ mol^–1^ (298 K) to −23 kJ mol^–1^ (700 K).

When the ΔGr of individual reaction steps and their corresponding side steps is compared, it can be concluded that the probability of butanol formation will be very small if thermodynamics is considered. The ethanol dehydrogenation (1st) is less preferred than the competing dehydration, which is even more pronounced with elevated temperatures. The acetaldehyde condensation (2nd) is less preferred over the ethyl acetate formation due to negative values of the ΔGr for ethyl acetate at every temperature. Similarly, for 3-hydroxy butyraldehyde dehydration (3rd), the formation of acetone should be preferable over dehydration at every temperature. On the other hand, the crotonaldehyde hydrogenation to 1-butanol (4th) was preferable over the hydrogenation to butyraldehyde. Based on calculated ΔGr, it is concluded that the reaction temperature as a significant factor should be considered together with properties of the catalyst to discuss the transformation of alcohols via the Guerbet reaction and abundance of side products.

#### 3.2.2 Catalytic Tests

The transformation of ethanol was conducted at 573, 623, and 673 K for all Cu/Li-Al catalysts. The catalytic experiment temperatures were selected based on previous tests presented in earlier publications,^7,19^ where the setting of reaction conditions was discussed in detail. Ethanol conversion (Figure 7) and selectivity of the products 1-butanol, acetaldehyde, isopropyl alcohol, butyraldehyde, ethyl acetate, butyl acetate, and hexanol were determined (Table 2). Conversion and selectivity were derived by averaging the results from a total of 7 reaction mixture samples at every temperature.

**Figure 7:**
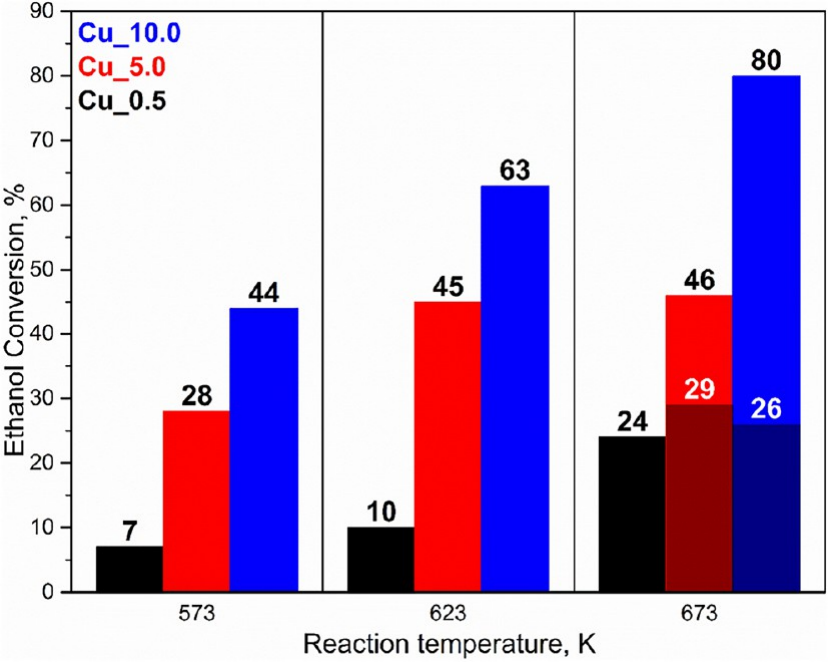
Ethanol conversion for Cu_0.5, Cu_5.0, and Cu_10.0 at 573, 623, and 673 K.

**Table 2:** Overview of Product Selectivity and Related Ethanol Conversions

	Cu_0.5, %						
temperature, K	*X*_EtOH_	*S*_Acetald_	*S*_EthAcet_	*S*_Acetone_	*S*_Butyr_	*S*_ButOH_	*S*_HexOH_
573	7	1	12	0	0	**14**	0
623	10	3	6	0	5	**20**	0
673	24	5	3	0	2	**20**	1

	Cu_5.0, %						
temperature, K	*X*_EtOH_	*S*_Acetald_	*S*_EthAcet_	*S*_Aceton_	*S*_Butyr_	*S*_ButOH_	*S*_HexOH_
573	28	24	7	0	11	**31**	1
623	45	16	7	0	13	**28**	5
673	29–46	6–15	3–5	0	2–11	**15–30**	1–2

	Cu_10.0, %						
temperature, K	*X*_EtOH_	*S*_Acetald_	*S*_EthAcet_	*S*_Aceton_	*S*_Butyr_	*S*_ButOH_	*S*_HexOH_
573	44	12	6	2	8	**28**	0
623	63	9	5	2	13	**30**	2
673	26–80	3–9	2–4	1–2	3–16	**14–28**	1–5

The detected ethanol conversion was in a wide range from 7 to 80%. Factors such as the catalyst composition and reaction temperature were observed. Among the catalysts studied, the most active catalyst was the catalyst with the highest copper loading, Cu_10. The reaction temperature positively influenced ethanol transformation as higher temperatures resulted in increased conversion. For Cu_0.5, ethanol conversion gradually increased with the reaction temperature, reaching 24% at 673 K. Cu_5.0 and Cu_10.0 related to the initial conversion increase (573–623 K) with a subsequent decline (673 K). In particular, Cu_5.0 reached 45% conversion at 623 K, and Cu_10 reached 80% conversion at 623 K. TOS data (Figure S3) showed the stabilization of conversion in time, for individual reaction temperatures. Up to a temperature of 623 K, the reaction stabilized relatively quickly (up to 5 h) after the temperature change, for all studied catalysts. The conversion value presented in Figure 7 is the average of the stabilized values. At the highest reaction temperature of 673 K, the conversion value was stable only for sample Cu_0.5, and for samples Cu_5.0 and Cu_10, a gradual decrease in conversion over time was observed (range of conversions in Figure 7). Obviously, the higher copper loading in Cu_5.0 and Cu_10 was associated with higher conversion due to the higher amount of dehydrogenation sites. However, a marked decline in conversion was observed at a higher temperature of 673 K for Cu_5.0 and Cu_10. After a few hours, there was practically no difference in conversion among the tested catalysts at 673 K. The observed phenomena were due to several factors. Structural changes were observed on XRD for Cu_5.0 and Cu_10, copper migration was observed on EDX and H_2_-TPR, and additionally, a larger amount of organic deposits was detected. The change in copper distribution and its poorer accessibility compared to the fresh catalyst contributed to the catalyst deactivation at the high reaction temperature of 673 K. In the case of Cu_0.5, the lower copper content led to the highly dispersed copper particles with the higher interactions of the Li-Al matrix, which relates to stable particles even at 673 K. In general, increased copper content led to improved ethanol conversion, but it was associated with the poorer usability of copper on the surface of the Li-Al mixed porous oxide. Additionally, the spent catalyst related to relatively lower CBS and CAS, which could also participate in ethanol conversion.^7,47^ The strong basic sites, which could dehydrogenate ethanol at higher temperatures, decreased in population for spent catalysts. In previous studies on Li-Al loaded with almost 38 wt % copper,^19^ the ethanol conversion was lower than that of Cu_5.0 due to the large (bulk) copper particles and deterioration of the catalyst surface. The observation highlights the significance of stabilizing copper as a crucial element in attaining high ethanol conversion over the long reaction period and a high WHSV. Conversely, another approach might involve the synthesis of catalysts that exhibit greater dehydrogenation activity at reduced temperatures.

A direct comparison of obtained ethanol conversion with data from the literature on individual catalysts is challenging since the reaction conditions vary a lot. In particular, the homogeneous^48^ or heterogeneous catalysis^49^ (the reaction mixture in liquid or gas phases), varying temperatures, pressure, and WHSV (catalyst load) are used. For illustration, selected data are presented in Table 3. For a relevant comparison of selectivity and the related catalyst performance, there is a need for isoconversion data obtained under at least similar reaction conditions. A relatively comprehensive comparison of catalysts can be found in the literature.^43^ The impact of the WHSV on catalytic results is rather omitted in the discussions. Guerbet transformations in flow bed reactors are frequently studied at a lower WHSV compared to the present study. In general, lower values of the WHSV, i.e., catalyst loads, correspond to increased values of ethanol conversions. For a low WHSV, in the limiting case, all of ethanol may react, and it is difficult to assess the activity among catalysts. At a given temperature, a low WHSV combined with low conversion indicates poor catalysts. At higher WHSVs, the difference in catalytic activity (conversion) would be pronounced due to the lower residence time.^50^ Additionally, for high WHSVs, potential catalyst degradation is likely to be applied, which can be described in shorter catalytic experiments. It has to be noted also that a higher WHSV relates closer to the conditions in the large scale of the chemical industry for production of large quantities of higher alcohols, which could be used in many ways, for example, as feedstock, fuel, plasticizers, etc.^4,51^ With the increasing WHSV and therefore a lower contact time, there is less production of higher alcohols when we focus primarily on the production of butanol as the main product. Cu/Li-Al mixed metal oxides used in this study are supposed to perform above average. The main reason is the high WHSV of 4.5 used, i.e., high loading of catalysts with ethanol feed, which could lower the final ethanol conversion compared to the other catalysts tested at low WHSVs (0.032–0.99). Other than that, similar trends were observed, such as an enhanced ethanol conversion with the reaction temperature and copper loading to a certain extent.

**Table 3:** Performance of Selected Catalysts under Variable Reaction Conditions

catalyst(s)	reaction phase	temperature, K	pressure, MPa	WHSV, h^–1^	*X*_EtOH_ at max, %	refs
Cu-Mg-Al	gas	598	2.07	0.20	65	^49^
Cu-M/Graphite (M = MgO, BaO, ZnO, MnO)	gas	503	0.5	0.03	30	^52^
Mg-Al-PMO^a^Ni_20_-PMO Cu_20_-PMO Cu_10_Ni_10_-PMO	condensed/gas	453–583	0.8	0.99	70	^53^
Ni/La_2_O_3_/Al_2_O_3_	condensed	483–543	-	0.8–2.1	42	^54^
Cu/LaPO_4_ Cu/CePO_4_ Cu/AlPO_4_	vapor	473–573	0.1–2	0.14	74.5	^55^

a PMO: porous metal oxide.

The reaction mixture contained a variety of products, while 1-butanol was considered as the desired (main) product. Other noteworthy products, such as ethyl acetate, acetone, butyraldehyde, or hexanol, were also present in the reaction mixture but at lower selectivities compared to 1-butanol. Other chemical compounds were also observed, but at selectivities close to 1% (diethyl ether, isopropyl alcohol, 1,1-diethoxy ethane, 1-pentanol, 2-ethyl-1-buthanol); therefore, they will not be discussed further. In general, products were related to decreased selectivity as the ethanol conversion increased with elevated temperatures (Table 2). The carbon balance as the percentage of carbon (hydrocarbons) found in the reaction mixture (liquid and gas) from the carbon introduced into the reaction in ethanol feed is displayed in Figure S4.

The theoretical calculations foreshadowed the reaction path without catalysts pointing to the Guerbet reaction as an unlikely reaction path based on thermodynamics (Figure 6) since ethanol dehydration to diethyl ether or ethylene was thermodynamically favorable over the dehydrogenation to acetaldehyde.

The presence of a catalyst affects the reaction rate and alters preferred reaction pathways. The previous publications could serve as an illustrative example,^7,19^ where the catalyst controlled the direction of the reaction. Lewis acid catalysts supported the ethanol dehydration pathway; meanwhile, basic catalysts with redox properties supported the ethanol dehydrogenation pathway. The experiments without a catalyst proved it was nearly impossible or extremely ineffective to realize the Guerbet reaction due to the high activation barrier and very high temperatures needed. To prioritize the dehydrogenation of ethanol to acetaldehyde, a relevant catalyst needs to be utilized.

Catalyst Cu_10.0 was the most active among studied catalysts (Figure 7; Table 2); therefore, the effects of catalysis on the reaction pathway will be demonstrated just on Cu_10.0. Utilizing catalyst(s) led to enhanced acetaldehyde formation over ethylene or its precursor diethyl ether. The selectivity of acetaldehyde reached values in the interval from 3 to 12% (Table 2); meanwhile, the selectivity of ethylene or diethyl ether was almost 0%. The shift to acetaldehyde could be explained by the presence of the redox properties, which lowered the activation energy for the dehydrogenation of ethanol to acetaldehyde. The dehydration would occur in the reaction if catalysts with dominant acidic properties were used (such as Al_2_O_3_).^19,56^

The aldol condensation of acetaldehyde to 3-hydroxy butyraldehyde was also in thermodynamical disadvantage compared to the side reaction to ethyl acetate (Tishchenko’s reaction). This side reaction has more potential to the ethylene side reaction because the selectivity of ethyl acetate reached values from 2 to 6% (Table 2). Almost no 3-hydroxy butyraldehyde was detected in the reaction mixture. Nevertheless, the absence of 3-hydroxy butyraldehyde could be explained by the swift reaction over crotonaldehyde to the final product (butanol), which was in the reaction mixture in a significant amount. The preference of aldol condensation over the ethyl acetate side reaction was related to the presence of catalysts with basic properties.

Similarly, the dehydration of 3-hydroxybutyraldehyde to crotonaldehyde was thermodynamically less favorable compared to the side reaction to acetone, which was detected in the reaction mixture with a selectivity up to 2% (Table 2). Also, crotonaldehyde was not detected in the reaction mixture, which could be explained as in the same way to 3-hydroxy butyraldehyde, i.e., the reaction of crotonaldehyde to 1-butanol is rapid and 1-butanol was found in the reaction mixture, which meant that crotonaldehyde was in the reaction as an intermediate.

The hydrogenation of crotonaldehyde to 1-butanol as the last step in the Guerbet reaction was in thermodynamical advantage over the side reaction to butyraldehyde. The higher selectivity to butanol (up to 30%), in comparison with butyraldehyde selectivity (up to 16%) (Table 2), corresponded to the theory based on the calculations. 1-Hexanol formation could serve as the confirmation that the Guerbet reaction can also produce even higher alcohols from ethanol (next to 1-butanol) despite the low selectivity in the range of 1–5% (Table 2). Another proof could be the presence of butyraldehyde, which serves as an intermediate product to 1-hexanol, and similarly acetaldehyde, which is the intermediate product to 1-butanol.

Combined theoretical calculations and experimental data showed that the reaction path could be significantly altered by engineering of catalyst(s) with appropriate properties. Despite the low feasibility given by reaction Gibbs energies of individual steps, bifunctional Cu/Li-Al catalysts allowed the Guerbet reaction by the combination of their redox and acid–base properties. The altered reaction path was already demonstrated in previous studies,^19,57^ which dealt with the catalysts based on mixed metal oxides having a wide range of acid–base and redox properties. Acidic catalysts promoted ethanol dehydration, while catalysts with higher basicity promoted the Guerbet reaction (ethanol dehydrogenation), and acid–base catalysts with redox properties promoted the Guerbet reaction on a greater scale, even at low temperatures. In present study, Cu/Li-Al catalysts were specifically tailored to study the influence of copper loading on the previously optimized Li-Al oxide matrix.^19^ The impregnation method for Cu/Li-Al preparation enabled a reproducible method of catalyst preparation. Therefore, the comparison of not only the ethanol conversion was more reliable but also the change in selectivity could be closely related to the copper loading. Even though the Cu loading related to a lower amount of CBS on the Cu/Li-Al surface (Table 1) (both fresh and spent catalysts) and a lower specific surface area, the selectivity to 1-butanol was relatively high for Cu_10 pointing to the sufficient density of sites on the surface. The Cu_5 and Cu_10 catalysts had practically the same selectivity to 1-butanol. The selectivity of acetaldehyde and 1-hexanol corresponded to the conversion of ethanol being relatively higher on the Cu_5 and Cu_10 catalysts. The higher selectivity to ethyl acetate for Cu_0.5 was related to the higher concentration of Lewis acid sites on that catalyst.

Despite the wide variety of reaction conditions (temperature, pressure, phase of the reaction mixture, etc.), similar selectivity results were previously observed,^58,59^ i.e., the order of Guerbet reaction products and side products selectivity with butanol (alcohols) being the main product(s) followed by esters (ethyl acetate), aldehydes (ethanal), etc. Production of esters could be considered as the major disadvantage since copper was reported to support this side reaction, which could make the elimination of this problem hard. On the other hand, specifically selectivity to ethyl acetate had a tendency to decrease with the increasing reaction temperature and ethanol conversion, but other side products had a tendency to be populated. One of these side products was butyraldehyde, which could originate from the hydrogenation of crotonaldehyde (alternation of the reaction path) or the dehydrogenation of 1-butanol (further reaction). Either way, butyraldehyde could be considered as an unwanted side product if the aim was 1-butanol. The other similarity was in the 1-butanol selectivity, which was in the interval from 30 to 35%, with the slight exception of 74% overall alcohol selectivity.^49^ Therefore, a possible steady state related to 30% butanol selectivity could be considered and should be further studied.

## 4 Conclusion

The Guerbet reaction of ethanol was investigated by combining quantum chemical calculations and practical long-term catalytic tests. The obtained data underscored the significant complexity of catalysis when considering a single-step approach, such as performing the Guerbet reaction within a single reactor. Calculated reaction Gibbs energies revealed the feasibility of reaction steps involved in the Guerbet reaction and main side reactions, highlighting the need for higher temperatures for effective dehydrogenation, while lower temperatures are preferred for subsequent reactions such as aldol condensation. The importance of the catalysis is primarily for accelerating reaction steps that are already thermodynamically feasible at higher temperatures. High conversions of ethanol to acetaldehyde are achieved at high temperatures, where side reactions are feasible, which supports alternative pathways related to the lower selectivity toward butanol. The ethanol conversion would exceed 80%, while butanol selectivity reaches up to 30%. Among the catalysts studied, the Li-Al mixed metal oxide with around 10 wt % impregnated copper related to the best catalytic performance. Ethanol conversion increased with copper loading to some extent; nevertheless, the highly loaded catalyst suffered from severe deactivation at temperatures over 623 K and a pressure of 10 MPa. Conducting the reaction in a single reactor under fixed conditions favors either the first and third steps or the second and fourth steps of the Guerbet reaction. Dividing the process into two separate reactors could offer a promising alternative. This approach allows for tailoring reaction conditions and catalyst properties to the specific requirements of each step, potentially resulting in a higher overall conversion rate and an improved selectivity of desired products.

## Supplementary Material



## Notes

EDX of fresh and spent catalysts (Figure S1); N_2_ physisorption on the spent catalyst (Figure S2); TOS data for catalytic tests (Figure S3); carbon balance for catalysis outcome (Figure S4); validation of theoretical values of reaction Gibbs energies (Table S1); and composition of the catalyst particle surface from EDX (Table S2) (PDF)

## Nomenclature

CO_2_-TPD: temperature-programmed desorption of CO_2_
H_2_-TPR: temperature-programmed reduction by H_2_
ICP-MS: inductively coupled plasma with mass spectrometry
LDH: layered double hydroxide
NFDLT: nonlocal density functional theory
ΔGr: reaction Gibbs energy
NH_3_-TPD: temperature-programmed desorption of NH_3_
TCD: thermal conductivity detector
XRD: X-ray diffraction analysis
wt %: weight percent(s)
WHSV: weight hourly space velocity, h^–1^
