# Supplementary material for: Conversion of Ethanol to Guerbet Alcohols and Other Products: Combined Catalytic and Theoretical Study
Source: ACS Omega. 2025 Dec 10;10(50):61308–21. doi: 10.1021/acsomega.5c05882 (PMC12750243; doi:10.1021/acsomega.5c05882)
Supplement: Supplementary file 1 [file ao5c05882_si_001.pdf]

Supporting Information for

Conversion of ethanol to Guerbet alcohols and other products: combined catalytic and theoretical study

Jan Malina[1], Karel Frolich[1], Martin Hájek[1], Jaroslav Kocík[2], Vladimír Lukeš[3], Erik Klein[3]*

[1]University of Pardubice, Faculty of Chemical Technology (Department of Physical Chemistry), Studentská 573, 532 10 Pardubice, Czech Republic

[2]ORLEN Unipetrol RPA s.r.o., Záluží 1, 436 70 Litvínov, Czech Republic

[3]Institute of Physical Chemistry and Chemical Physics, Slovak University of Technology in Bratislava, Radlinského 9, Bratislava SK-812 37, Slovakia

**Corresponding author: karel.frolich@upce.cz*

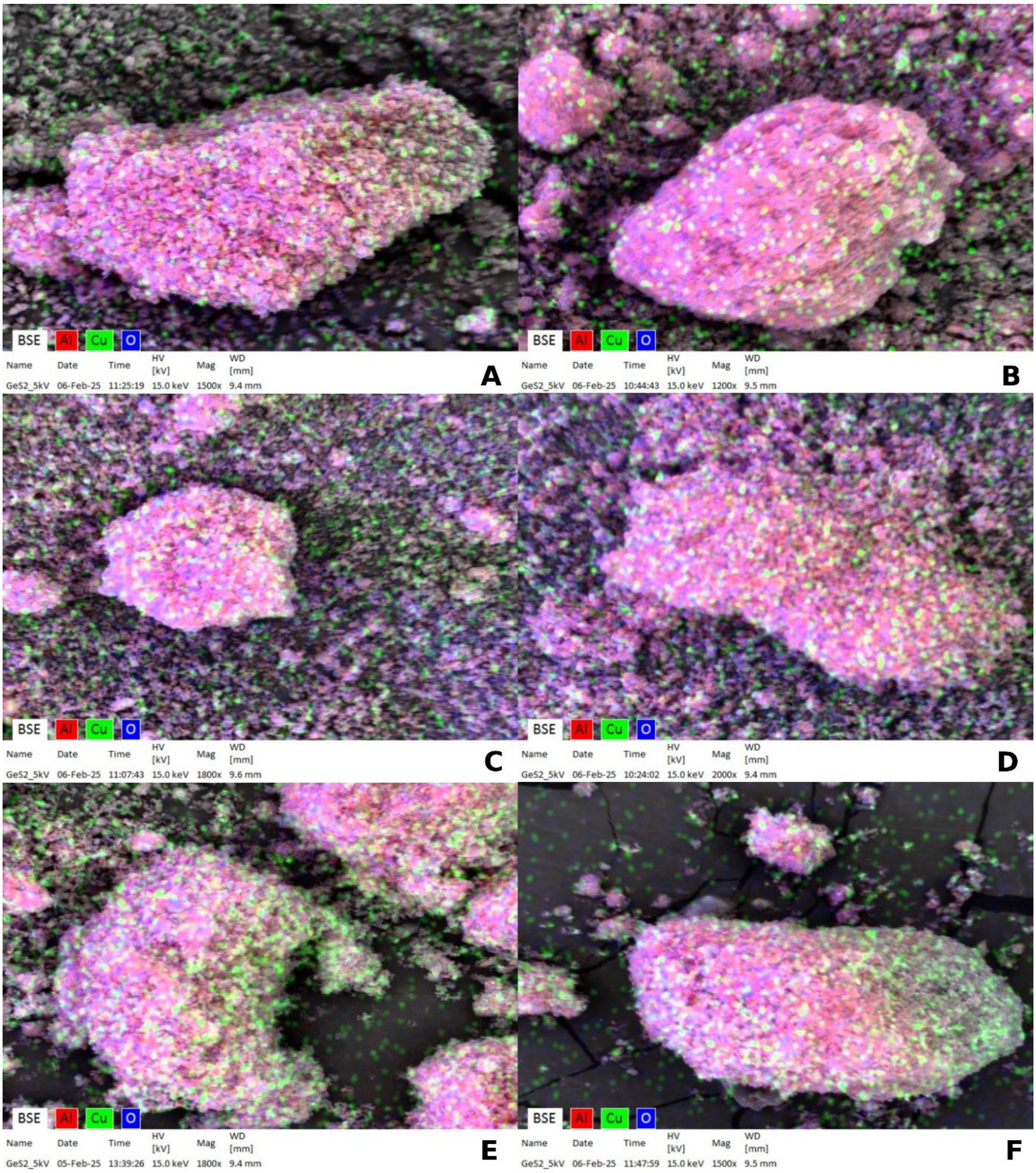

Figure S1 EDX of fresh (left side) and spent (right side) catalyst. Cu_0.5 A) and B), Cu_5.0 C) and D), Cu_10.0 E) and F)

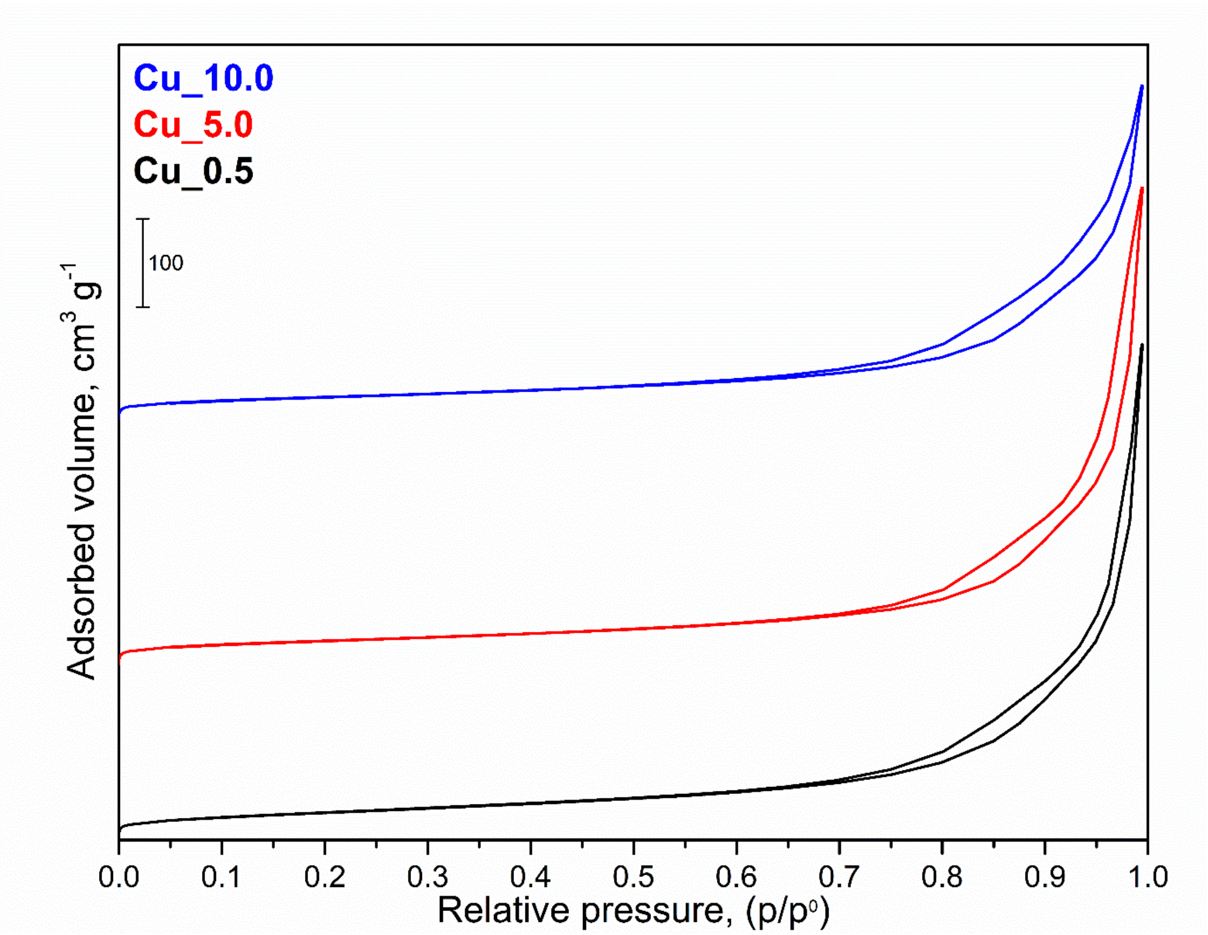

Figure S2 N_2-physisorption on Cu_0.5, Cu_5.0 and Cu_10.0 spent catalysts; corresponding S_{BET} and pore volumes in Table 1

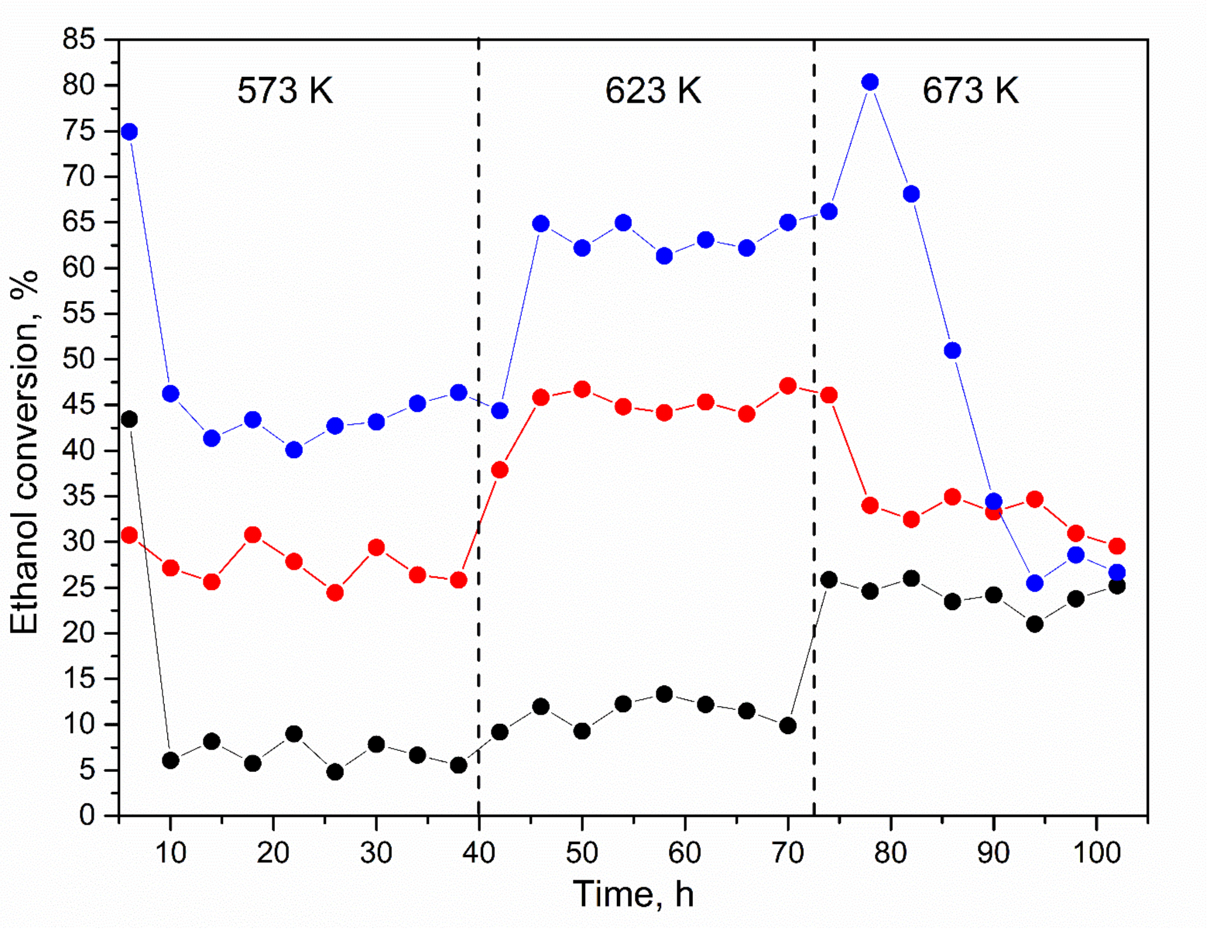

Figure S3 Time on stream (TOS) for catalytic tests (outlayers were excluded from the average values presented in Figure 7); Cu_0.5 (Black), Cu_5.0 (Red) and Cu_10.0 (Blue)

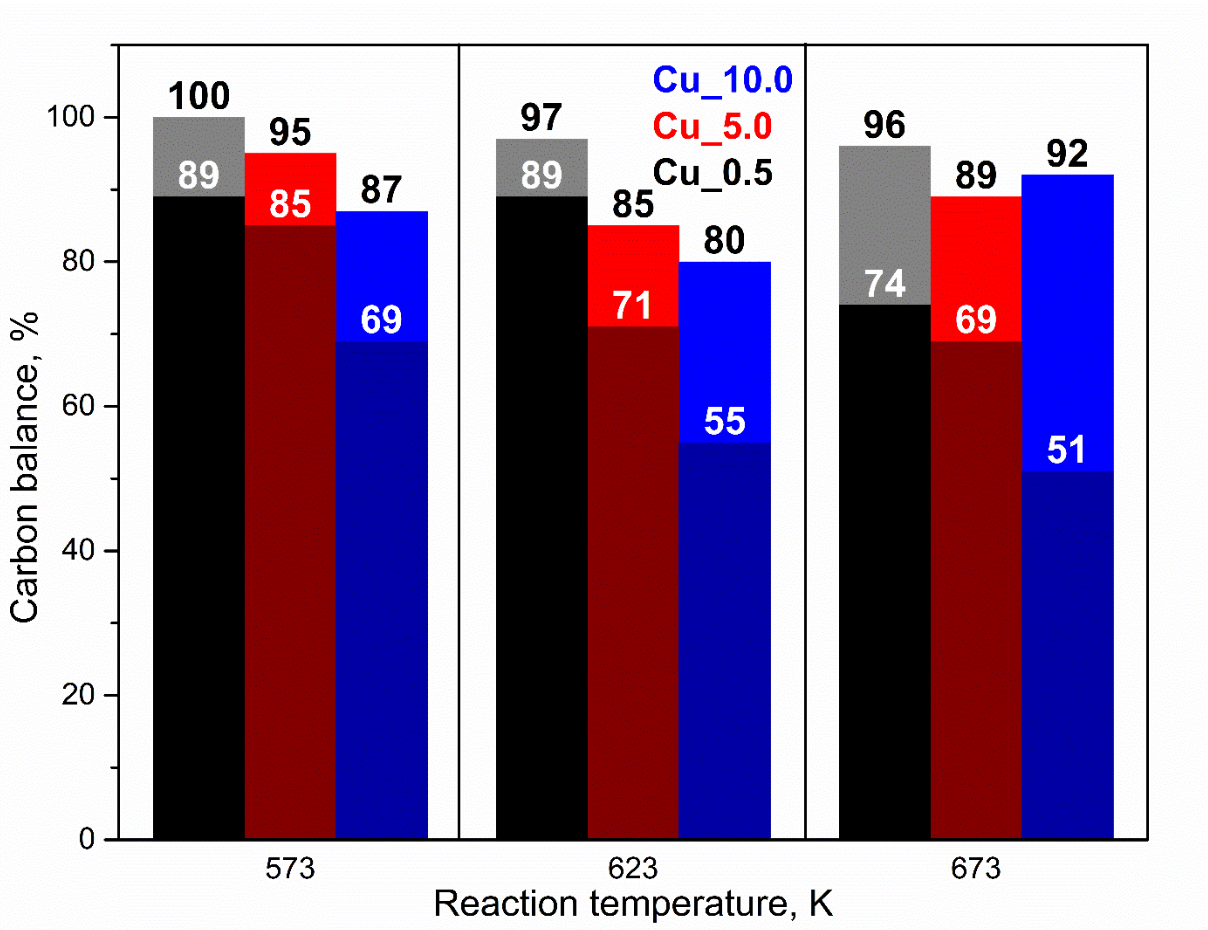

Figure S4 Carbon Balance: percentual expression of carbon (hydrocarbons) found in reaction mixture (liquid and gas) from carbon introduced to the reaction in ethanol feed

Additional information: The carbon balance was calculated from the detected and determined components (liquid and gaseous). The remainder up to 100% consists of substances that were either not determined or captured (volatile components and gases), the amount of which was proportionally higher at higher temperatures. There was no on-line detection, the reaction mixture samples were taken after the reactor and transferred to chemical analysis. Detected but not determined substances were related to the very low response.

Table S1 Validation of theoretical values of reaction Gibbs energies.

Reaction	$\Delta_r H^\circ$, kJ mol^{-1}	
	Exp.	M06-2X
C_2H_5OH ® $CH_3CHO + H_2$	70	74
CH_3COOH ® $CO_2 + CH_4$	–35	–27
$CH_3CH{=}CH_2 + H_2$ ® $CH_3CH_2CH_3$	–125	–131
$CO + H_2O$ ® $CO_2 + H_2$	–41	–46
$CH_4 + H_2O$ ® $CO + 3H_2$	206	203
$2\ CH_4 + CO_2$ ® $CH_3COCH_3 + H_2O$	84	81
$CH_3CHO + CO_2$ ® $CH_3COOH + CO$	16	13
$2CH_4$ ® $H_2C{=}CH_2 + 2H_2$	201	204
$4CH_4$ ® $C_4H_{10} + 3H_2$	172	164

Table S2 Composition of catalyst outer surface from EDX in %

Catalyst	C	O	Al	Cu
Cu_0.5 – Fresh	-	64.56	33.96	1.47
Cu_0.5 – Spent	14.92	56.28	28.57	0.23
Cu_5.0 – Fresh	-	66.08	31.35	2.58
Cu_5.0 – Spent	30.41	49.85	18.52	1.22
Cu_10.0 – Fresh	-	63.61	30.56	5.83
Cu_10.0 – Spent	26.48	52.22	19.44	1.86
